# Rhombencephalitis in Pregnancy—A Challenging Case of Probable *Listeria* Infection

**DOI:** 10.3390/life12101600

**Published:** 2022-10-14

**Authors:** Alison E. P. Ho, Zahirrah B. M. Rasheed, James Norman, Carolyn Gabriel, Luke Dixon, Simon Ashworth, Charlotte Frise, Christina K. H. Yu, Lynne Sykes

**Affiliations:** 1 St. Mary’s Hospital, Imperial College Healthcare NHS Trust, London W2 1NY, UK; 2 King’s College Hospital, NHS Foundation Trust, London SE5 9RS, UK; 3 UKM Medical Molecular Biology Institute (UMBI), Universiti Kebangsaan Malaysia, Kuala Lumpur 56000, Malaysia; 4 Imperial College Parturition Research Group, Department of Metabolism, Digestion and Reproduction, Imperial College London, London SW7 2BX, UK; 5 Queen Charlotte’s and Chelsea Hospital, Imperial College Healthcare NHS Trust, London W12 0HS, UK; 6 The Parasol Foundation Centre for Women’s Health and Cancer Research, St. Mary’s Hospital, Imperial College Healthcare NHS Trust, London W2 1NY, UK

**Keywords:** *Listeria monocytogenes*, rhombencephalitis, pregnancy

## Abstract

Rhombencephalitis refers to inflammation of the brainstem and cerebellum, and can be caused by infections, autoimmune disorders or paraneoplastic syndromes. The most common infective cause is the bacterium *Listeria monocytogenes.* *Listeria monocytogenes* is the predominant species to cause human listeriosis, and is commonly due to the ingestion of contaminated foods. Symptoms include a mild gastroenteritis, fever (often with extreme temperature variations), headache, and myalgia. In more severe cases, invasive disease may lead to bacteraemia and neurolisteriosis. Pregnant women are more susceptible to listeriosis, which is believed to be due to pregnancy-related immune modulation. Maternal-neonatal infection with adverse pregnancy outcomes include neonatal listeriosis, spontaneous miscarriage and intrauterine fetal demise. Diagnosis may be challenging due to initial nonspecific symptoms and low sensitivity and specificity of confirmatory diagnostic laboratory tests. Here, we describe a case of rhombencephalitis in pregnancy, attributed to *Listeria*, and review the clinical features, diagnosis and multidisciplinary management. Lastly, we describe the immunological response to *Listeria monocytogenes* and show in vitro pro-inflammatory effects of *Listeria monocytogenes* on peripheral blood mononuclear cells and placental explants.

## 1. Introduction

*Listeria* is a Gram-positive bacillus and facultative anaerobic motile rod. It can be found in soil, water, processed foods, raw meat and the faeces of both animals and humans. It is estimated that 1–5% of the general population are asymptomatic carriers [1]. *Listeria monocytogenes (LM)* is one of 20 *Listeria* species and is the predominant one to cause human listeriosis, which is commonly due to ingestion of contaminated food such as paté, soft cheeses, and unpasteurized dairy. It may result in a mild gastroenteritis, fever (often with extreme temperature variations), headache, myalgia and sore throat. In more severe cases, it leads to invasive disease, manifesting as; bacteraemia, neurolisteriosis, and/or maternal-neonatal infection. Invasive disease is associated with high maternal and fetal mortality.

Pregnant women are more susceptible to listeriosis most likely due to the immune modulation that occurs in pregnancy, with a reported incidence of 3.4–16.69 per 100,000 [2,3,4,5] compared to 0.2–0.5 per 100,000 in the general population [6]. Pregnancy complications of listeriosis include miscarriage, stillbirth, and spontaneous preterm labour with meconium-like stained amniotic fluid. Chorioamnionitis and vertical transmission to the neonate may occur, manifesting as neonatal pneumonia, sepsis, meningitis, or in some cases granulomatous infantiseptica with widespread microabscesses and granulomas. Neonatal listeriosis has a fatality rate of 20–60% and has a reported incidence of 8.6 per 100,000 live births [7].

Severe cases of listeriosis can result in neurological complications including meningoencephalitis, rhombencephalitis (inflammation of the brainstem and cerebellum), cerebritis, intracranial abscesses, and death. Here, we describe a case of rhombencephalitis attributed to *Listeria* in pregnancy, and review the clinical features, diagnosis, and management. We also summarise pregnancy-specific adverse effects and immune responses relating to listeriosis and provide in vitro evidence to support the mechanism of immune mediated adverse pregnancy events.

## 2. Case Presentation

A 31-year-old woman presented at 21 weeks of gestation in her first pregnancy with a fever of 38.0 °C and a four-day history of frontal headache. There was no associated nausea, photophobia, neck stiffness, rash, visual disturbance, cough or sore throat. There was no history of recent foreign travel, or of eating raw meat or unpasteurised food. Neurological examination was initially unremarkable. Blood tests performed revealed a C reactive protein (CRP) of <5 mg/L (normal), white cell count (WCC) of 11 × 10^9^/L (normal for pregnancy), neutrophil count of 9.0 × 10^9^/L (mildly raised) and lymphocyte count of 1.3 × 10^9^/L (normal). Her routine antenatal screen for HIV, Hepatitis B and syphilis were negative. Her fever settled and the impression was that her symptoms were likely secondary to a mild viral illness. She was therefore discharged with analgesia and given advice to return if her symptoms persisted.

She returned on day eight with ongoing fevers, headache, general fatigue, and myalgia. Routine observations revealed a mild pyrexia of 37.5 °C with a normal heart rate of 69 bpm and blood pressure of 110/67 mmHg. All examination findings were unremarkable, and her CRP was <5 mg/L (normal) with a white cell count of 11.2 × 10^9^/L (normal for pregnancy), neutrophil count 9 × 10^9^/L (mildly raised), lymphocyte count 1.3 × 10^9^/L (normal), monocyte count 0.9 × 10^9^/L (normal), and lactate 1.1 mmol/L (normal). Due to concerns regarding pyrexia of unknown origin (PUO), blood cultures and urine culture were sent, with nasopharyngeal swabs were sent for an extended panel of respiratory viruses including SARS-CoV-2.

The patient was commenced on empirical intravenous antibiotics of ceftriaxone 2 g once a day and metronidazole 500 mg 8 hourly, in keeping with hospital guidelines for PUO. She continued to have a frontal headache and intermittent temperature spikes, with rapid fluctuations between 36.2 and 39.2 °C despite antibiotic treatment. Given the constellation of symptoms and ongoing headache, there was concern about intracranial infection, which led to increasing the ceftriaxone 2 g to twice daily, adding in amoxicillin 2 g 4 hourly alongside acyclovir dosed for intracranial infection, in this case 500 mg 8 hourly, specifically covering for *LM*, herpes simplex virus (HSV) I and II, and varicella zoster virus (VZV). Viral serology was sent for human immunodeficiency virus (HIV), cytomegalovirus, Epstein–Barr virus, varicella zoster, and Hepatitis A, B, C and E. Chest radiograph was normal. The diagnoses of haemophagocytic lymphohistiocytosis, familial Mediterranean fever and granulomatosis with polyangiitis were considered, but felt to be unlikely. Unenhanced magnetic resonance imaging (MRI) of her brain was normal with no evidence of intracranial infection, meningitis or encephalitis.

On day 13 she became confused, and her personality changed. Her abbreviated mental test score deteriorated to 8/10, yet there were no focal neurological deficits noted on examination. Her inflammatory markers remained normal (WCC 9.4 × 10^9^/L, CRP 5.1 mg/L). MRI of the brain and cervical spine were normal, and an MR venogram showed no evidence of a cerebral venous thrombosis. Cerebrospinal fluid (CSF) from lumbar puncture (LP) revealed a raised white cell count of 75/µL (with 75% mononuclear cells) (normal range 0–5/µL), red cell count of 1, protein 1.88 g/L (normal range 0.15–0.45 g/L), lactate 3.8 mmol/L (normal range 1.0–2.9 mmol/L); paired serum lactate of 0.84 mmol/L, glucose 2.1 mmol/L (normal range 1.8–4.7 mmol/L); paired blood glucose of 6.4 mmol/L. A bed-side electroencephalogram (EEG) was consistent with a non-specific generalised encephalopathy.

On day 14 she deteriorated further, and became more drowsy and less coherent, repeatedly closing her eyes during conversation. She was orientated to month and year but not day. She then had a witnessed seizure which lasted approximately 10 s with subtle upper limb rhythmic low amplitude jerking. On examination, she had a full range of eye movements with no nystagmus, no fatiguability and normal saccades. Plantars were downgoing though she had brisk reflexes throughout. Given her seizures, she was started on levetiracetam (2 g loading then 500 mg twice a day) which was subsequently switched to lacosamide in view of hyponatremia.

By day 15 the patient had become disorientated and drowsy, with intermittent dysconjugate eye movements. Glasgow Coma Score (GCS) fell to 10 (E3, V2, M5) and she was noted to be intermittently obstructing her airway with paradoxical chest wall and abdominal movements, accompanied by desaturation on air from 96% to 93%. Given concerns with her fluctuating GCS, she was transferred to the intensive care unit (ICU). Her antibiotics were changed to meropenem (ceftriaxone and amoxicillin were stopped), while acyclovir was continued. An urgent computed tomography (CT) of the was also performed, which showed no acute abnormality.

**Figure 1:**
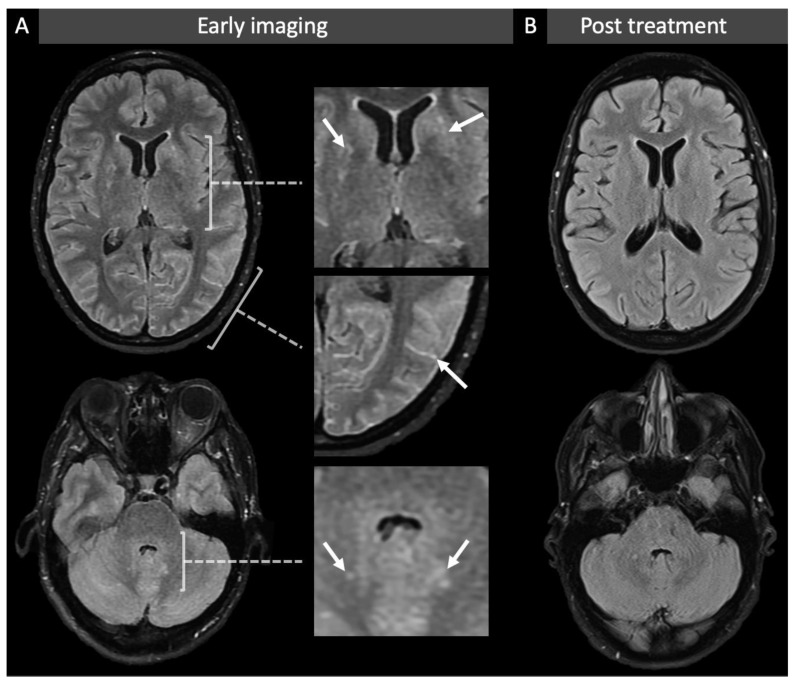
MR images before and after treatment. Panel (**A**): Axial MRI FLAIR images of the supratentorial (top row) and infratentorial (bottom row) brain at nadir (day 12 from symptom onset) demonstrate abnormal, ill-defined, high T2w signal in both basal ganglia (top magnified image) and dentate nuclei (bottom magnified image) in a perivascular distribution. Abnormal intra-sulcal FLAIR hyperintensity is also noted at this time (middle magnified image). Panel (**B**): Repeat axial FLAIR images following treatment (day 33 from symptom onset) demonstrate resolution of the abnormal signal in the deep ganglionic structures and sulcal spaces.

While in ICU, the patient developed intermittent right head turn with occasional myoclonic jerks. There was drooling from her right side of her mouth with no obvious facial weakness. There was no nystagmus. Her pupils were miotic and bilateral VI^th^ CN palsies developed on day 17. There was generalised hyperreflexia with finger jerks and 3 beats of clonus in her right ankle. Plantars remained downgoing. She developed a bilateral intention tremor but could follow a two-stage command. MRI was performed under general anaesthetic and showed new subtle T2-weighted hyperintensities in the basal ganglia, brainstem, and dentate nuclei with increased prominence to the deep perivascular spaces (Figure 1A). These findings were suggestive of an infectious basal meningitis with involvement of the brainstem and deep ganglionic structures, with both *cryptococcus* and *Listeria* postulated as potential pathogens. Fluid-attenuated inversion recovery (FLAIR) sulcal hyperintensity was also noted in both cerebral hemispheres which was felt to be either related to meningitis or iatrogenic secondary to factors such as oxygenation and propofol use. As a result of these findings, liposomal amphotericin B (5 mg/kg) was started while awaiting the results of a CSF cryptococcus antigen screen, and gentamicin (90 mg three times a day) was started to cover for suspected neurolisteriosis, dosing as per the British National Formulary (BNF). Amoxicillin was also restarted at a dose of 2 g 4 hourly to cover listeriosis. MRI of the thorax and abdomen showed no evidence of a tumour, so the diagnosis of a paraneoplastic phenomenon was felt to be less likely.

Extensive serological investigations were performed, none of which yielded any positive results (Table 1). Investigation for auto-immune encephalitis was negative for anti-N-methyl D-aspartate (NMDA) receptor, anti–leucine-rich glioma-inactivated 1 (Lgl1), anti-contactin-associated protein-like 2 (CASPR2), and anti-neuronal antibodies. Lymphocytes subsets and IgG subsets were normal. Auto-immune screen was negative. Repeat LP showed CSF 111 white cells/µL (100% mononuclear cells), protein 1.05 g/L (oligoclonal bands negative), glucose 2.7/5.6 mmol/L and lactate 3.2 mmol/L with no organisms seen on Gram-staining or identified on a range of antibody and antigen tests.

By day 19 the patient had improved clinically with an improving GCS and resolution of her bilateral VIth CN palsies. Acyclovir and liposomal amphotericin B were subsequently stopped with negative tests for HSV I and II, VZV, and Cryptococcus on the repeat LP, and she completed a 7-day course of gentamicin along with a 4-week course of intravenous amoxicillin. MRI shortly before completion of antibiotics showed improved intracranial appearances (Figure 1B) with two small focal lesions within the dorsal spinal cord suspected to be related to the resolving meningitic process.

**Figure 2:**
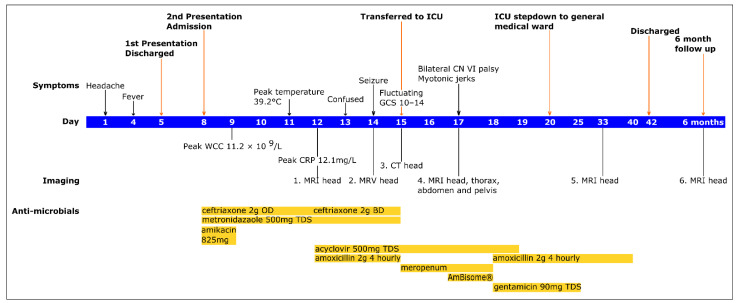
Timeline of symptoms and signs, imaging and antimicrobial administration. Key imaging results were: (1). MRI brain normal, (2). MRV brain no evidence of cerebral venous sinus thrombosis, (3). CT normal, (4). MR brain basal ganglia, brainstem and dentate nuclei abnormal signal and FLAIR sulcal hyperintensity with unremarkable thorax, abdomen, and pelvis, (5). MRI brain improved intracranial appearances with two small focal lesions within the dorsal spinal cord, (6). MRI brain almost complete resolution of the previously demonstrated abnormalities. The rationale for each imaging was as follows: (1). Day 12 MRI to determine a cause of her persistent frontal headache and confusion, (2). Day 14 MRV to determine if there was a venous thrombosis causing her headache, although acknowledgement this was of low suspicion, (3). Day 15 CT was performed due to her fluctuating glasgow coma scale, (4). Day 17 MRI head/thoracic/abdomen/pelvis was performed to rule out the possibility of a paraneoplastic syndrome and to see if there were any changes in the MRI head, given ongoing neurological symptoms, (5). Day 33 MRI head to determine response to treatment, (6). 6-month MRI head to confirm improvement. ICU (intensive care unit), CN (cranial nerve), WCC (white cell count), CRP (C-reactive protein), MRI (magnetic resonance imaging), MRV (magnetic resonance venography) CT (computerised tomography), OD BD TDS (Latin abbreviations referring to once, twice and three times a day respectively).

The patient was discharged on day 42 with physiotherapy, obstetric, and neurology follow up. On discharge she was able to walk but had a broad-based gait and required assistance of one person. Neurological clinical findings that remained were limb ataxia and intention tremor. Power was full, reflexes all present, with bilateral ankle clonus. A timeline of events is presented in Figure 2.

Following discharge, the patient developed vertigo and vestibular dysfunction suspected to be secondary to gentamicin, despite her levels having been mostly within target ranges during the course of her treatment. Audiological assessment confirmed her hearing was normal. She was monitored closely during the antenatal period. Serial fetal ultrasounds were reassuring with normal fetal growth and no obvious structural defects of the brain, liver, bowel, and placenta. She had no episodes of threatened preterm labour or preterm prelabour rupture of membranes and subsequently delivered at 39 weeks’ gestation by uncomplicated elective caesarean section. Reassuringly her baby was neurologically intact and investigations did not reveal any vestibular dysfunction or hearing impairment.

Follow-up MR brain at 6 months showed continued improvement with almost complete resolution of the previously demonstrated abnormalities. Her vestibular dysfunction improved with vestibular physiotherapy and her gait returned to normal. Given the patient’s clinical presentation, plus her extensive investigations, including characteristic MR brain imaging findings, the most likely diagnosis was felt to be rhombencephalitis secondary to *LM*.

## 3. Rhombencephalitis

*LM* is more likely to cause invasive disease such as bacteriaemia and central nervous system (CNS) infections in vulnerable populations such as those who are immunosuppressed, for example pregnant women, or those at the extremes of age. CNS listeriosis is rare, with a reported incidence of 7.4–16 cases per population of 1 million [8]. Entry to the CNS is thought to be via three potential routes: direct invasion of endothelial cells, transport across the blood–brain barrier within leukocytes, or migration within axons of the cranial nerves [9]. Sequelae of neurolisteriosis include meningoencephalitis, hydrocephalus, intracranial haemorrhage and rhombencephalitis [10].

The aetiologies of rhombencephalitis can be divided into infectious, autoimmune or paraneoplastic syndromes. The most common infectious cause is *LM*, followed by enterovirus 71, then herpes viruses. The most common autoimmune cause is Behçet’s disease (BD) [8]. Encephalitis secondary to *LM* was first described in the literature by Eck in 1957 [11]. He described the clinical course of the condition as one starting with a febrile illness, headache and vomiting with an abrupt onset of cranial nerve deficits and encephalopathy, and subsequent rapid deterioration with a fatal outcome. Later, in 1964, the syndrome of *Listeria* rhombencephalitis was described, featuring pontomedullary signs, hemiparesis and death from respiratory or cardiac failure [12].

### 3.1. Clinical Features and Diagnosis

The clinical features can help to differentiate aetiology of rhombencephalitis [8]. Fever is more common in infections and BD, but an infrequent feature of paraneoplastic disease. Cerebellar ataxia is more common in infections and paraneoplastic diseases and less frequently seen in BD. Altered consciousness is seen predominantly seen in those with infectious aetiology [8]. Our case had all three of these clinical features. *LM* is consistently reported to be the most common cause of rhombencephalitis. A series of 63 cases reports a typical prodromal illness of headache, nausea, vomiting and fever with a median duration of 4 days prior to abrupt onset of neurological features including dizziness, confusion, unsteadiness, weakness, and focal neurological signs [13]. Great variation in neurological signs is recognised with the most common being cranial nerve (CN) palsies in up to 100% of cases. The most frequent nerves affected are CN VI and VII. Other signs include pontomedullary and cerebellar abnormalities such as dysphagia, dyspnoea, dysarthria, ataxia, vertigo, and respiratory failure.

Importantly, the diagnosis of *Listeria* infection can be difficult to make. Despite fevers above 38 °C being described in the majority of patients, leukocytes remain below <15,000 cells/µL in 95% of cases. Confirmatory diagnostic laboratory tests are not always possible for various reasons, such as the intracellular location of *Listeria,* as well as the delayed testing following several days of empirical treatment. In our case, despite a negative PCR, it was acknowledged and reported by the laboratory team that low volume CSF can lead to a false negative result, and that PCR is insensitive for *Listeria*. CSF findings may be normal, but the most common abnormalities being a pleocytosis in 75% of cases, with literature reporting either equal percentages of mononuclear and polymorphonuclear cells, a predominance of mononuclear or a predominance of polymorphonuclear cells [8,14,15]. CSF protein is often normal, but can be high. Likewise CSF glucose is often normal, but can be low. The rates of positive CSF cultures are reported to be as low as 10–41% in CSF samples, and 61% in blood, even with repeated testing [13]. In one case series, despite 10% of cases having both negative CSF and blood cultures, a positive brain tissue culture was found at the time of autopsy [13]. Serological testing is also limited as antibodies to *Listeria* can be associated with exposure to highly prevalent non-pathogenic serotypes. Low sensitivity is encountered with serological testing, especially in early infection. Furthermore, specificity is low, due to antigenic cross reactivity with other Gram-positive bacteria such as *Staphylococci* and *Enterococci* [14,16,17,18].

Non-diagnostic peripheral blood results, with CSF and CT imaging in context of an evolving brainstem syndrome should raise suspicion of brainstem encephalitis secondary to *Listeria*. The investigation that is most likely to visualise abnormality is MRI. In a case series of 97 patients with rhombencephalitis, MRI abnormality was seen in 100% of cases attributed to *Listeria* [19]. On T2-weighted sequences, areas of hyperintense signals are seen in the brainstem, cerebellum and upper cervical spinal cord [20].

### 3.2. Treatment

Invasive listeriosis should be treated with intravenous aminopenicillin such as amoxicillin or ampicillin, or benzylpenicillin, and UK guidance recommend this should be given for a 21 day course in cases of neurolisteriosis [6]. The addition of gentamicin can lead to synergistic effects and has bactericidal properties. A large prospective study showed that addition of gentamicin reduced mortality when administered for 7 days (odds ratio of 0.6, *p* < 0.024) [21]. Our local antibiotic guideline advocated treatment with amoxicillin 2 g IV 4 hourly, and a seven-day course of gentamicin 90 mg 8 hourly (5 mg/kg divided into three doses/day) was also given following specialist advice.

A dramatic improvement in the clinical picture was only seen after commencing gentamicin, supporting the literature on its synergistic effect with combined penicillin treatment. Survival is between 70–80% when *Listeria* meningitis treated, compared to historic reports of 34% pre-antibiotic use [8]. In survivors the rate of lasting neurological deficits varies between 32 and 55%, which may reflect the heterogeneity of timing and type of antibiotic treatment [8,14]. The most common persisting deficits are cerebellar symptoms, limb motor weakness and VIIIth CN palsy [6].

## 4. Maternal-Neonatal Listeriosis

Pregnancy is a major risk factor for listeriosis with a crude incidence estimated to be between 10–100 times higher than the general population [22]. Maternal and neonatal consequences of listeriosis occurs following entry to the blood stream via lymphatic drainage of the intestine. Sequestration by immune cells can lead to release into the blood stream causing a secondary bacteriaemia, leading to its transportation to mucosal surfaces and the placenta. The MONALISA cohort study of 818 women with listeriosis showed that only 5% of pregnant women experienced uneventful pregnancies and delivery [21]. Lamont et al., reported on large series of listeriosis in pregnancy and found that although 29% of women were asymptomatic, 32% had a flu-like illness and 65% had a fever [23]. Gastro-intestinal (GI) symptoms are also frequently seen, and *Listeria* should be considered in pregnant women presenting with diarrhoeal illness. However, a diagnosis of *Listeria* should not be excluded in the absence of GI symptoms. Pregnancy-related adverse outcomes due to listeriosis include spontaneous fetal loss (10–20%), preterm delivery (45–50%), intrauterine fetal death (11–24%), 34% fetal distress (34%) and meconium stained liquor (75%) [21,23,24,25].

Vertical transmission can occur when *Listeria* is transmitted via haematogenous spread or via ingestion of contaminated amniotic fluid. Infections in early pregnancy are associated with spontaneous miscarriage, while those in the second or third trimester are associated with intrauterine fetal death, with rates as high as 26% [26]. Neonatal listeriosis occurs in approximately 8/100,000 live births with a mortality rate as high as 20% [24,26,27]. Other sequelae include neonatal sepsis and meningitis. Early-onset infection (days 1–7 of life) is associated with intrauterine infection and usually presents as premature delivery, in association with meconium-stained liquor, pustular lesions of the skin and pharynx, hypothermia and lethargy [28]. Late-onset (days 8–28 of life) is more likely to be as a result of birth canal or nosocomial transmission, and is more likely to be associated with the delivery of a healthy term baby, with development of sepsis or meningitis within the first month of life [29]. Long-term neurological damage in surviving offspring has been reported to be as high as 12.7% [23,25].

## 5. Immunological Response to *Listeria monocytogenes*

The innate and adaptive immune responses play a role in preventing *Listeria* associated disease, and is reviewed by Poulsen et al. [30]. Innate mechanisms provide the first line of defence, for example the low pH of the stomach, the intestinal microflora, and antimicrobial peptides. Additionally, innate immune cells will recognise *LM* via their receptors for pathogen associated molecular patterns (PAMPs). The pattern recognition receptors (PRR) Toll like receptors (TLR) 2 and 5 are able to recognise *LM* lipoteichoic acid and flagellin respectively. Recognition via these TLRs signal via myeloid differentiation factor 88 (MyD88) to prompt macrophages to produce cytokines like IL-1, IL-6 and TNF-α. This, in turn, can activate the arachidonic acid inflammatory cascade to produce leukotrienes which attract neutrophils. The adaptive immune response plays a key role in a more specific capacity, with CD8+ cytotoxic T cells directing cell mediated clearance. Activated CD4+, CD8+, and natural killer cells produce IFN-γ, which differentiate CD4+ T-helper cells to the T helper 1 (Th1) phenotype. This in turn this leads to further expansion of CD8+ T cells and increases MHC class 1 and class II antigen presentation. *LM* has an intracellular life cycle, thereby facilitating an effective evasion strategy from the host immune response. The bacterium can penetrate cells directly and pass between cells without accumulating in the extracellular space. This means the bacteria can easily evade humoral components of the immune response such as complement, cytokines, antibodies, and innate immune cells such as neutrophils. Since clearance is predominantly via T cell mediated immunity, host resistance and immunisation against listeriosis comes from sensitised lymphocytes and not from serum specific antibodies.

Conditions with reduced cell-mediated immunity are related to increased susceptibility to listeriosis. The reduction in cell-mediated immunity that occurs in pregnancy is a crucial immunomodulatory strategy to facilitate the growth of the semi-allogenic fetus. A well-cited concept is the recognised shift away from a Th1 response (responsible for cell mediated immunity), towards a Th2 response (humoral response) [31]. Despite recent belief that this is an oversimplification of the immune modulation that occurs in pregnancy, there is undoubtably an increase in susceptibility to intracellular pathogens such as *LM*, *Toxoplasma gondii* and viruses such as herpes, all of which rely on cell-mediated immune responses to prevent disease. In contrast, pregnancy is not considered to be a risk factor per se for maternal neurolisteriosis [22]. Although maternal neurolisteriosis is extremely rare, to date it is largely reported in pregnant women with immunosuppressive comorbidities [21,22]. Despite thorough investigation, we did not find any other evidence of immunosuppression in this case.

## 6. In Vitro Studies of the Effects of *Listeria* on Gestational Tissue

**Figure 3:**
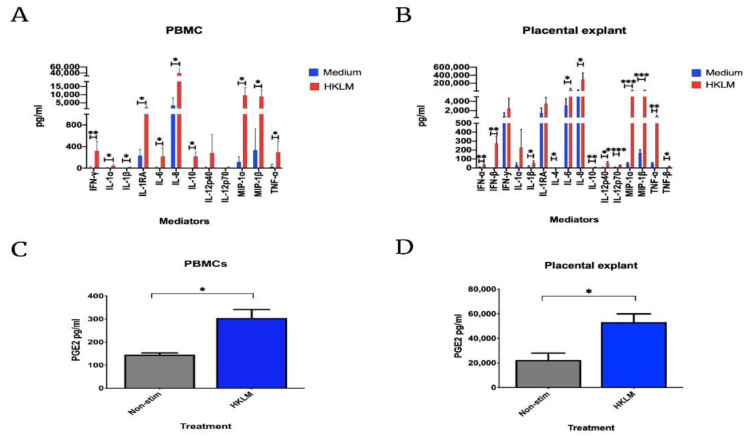
Peripheral blood mononuclear cells (PBMCs) and placental explant effects with heat-killed *Listeria monocytogenes* (HKLM) stimulation. PBMCs and placental explants were stimulated with 10^8^ of HKLM agonist for 18 h in PBMCs and 24 h in placental explant. The supernatant was used to detect 16 panels of inflammatory cytokines and PGE2. In PBMCs (**A**), IFN-γ, IL-1α, IL-1β, IL-1RA, IL-6, IL-8, IL-10, MIP-1α, MIP-1β, and TNF-α were significantly increased compared to the vehicle. A similar increase was also observed in the placental explant (**B**) with IFN-α, IFN-β, IL-1β, IL-4, IL-6, IL-8, IL-10, IL-12p40, IL-12p70, MIP-1α, MIP-1β, TNF-α, and TNF-β were statistically significant compared to the vehicle. PGE2 in both PBMCs (**C**) and placental explant (**D**) also significantly upregulated compared to the non-stimulated control cells. * *p* < 0.05, ** *p* < 0.01, *** *p* < 0.001, **** *p* < 0.0001. Non- stim (non-stimulated), HKLM (heat-killed *Listeria monocytogenes),* peripheral blood mononuclear cells (PBMCs), PGE2 (prostaglandin E2).

The association between maternal-fetal listeriosis and preterm delivery is poorly understood [32], however it has been suggested from two case reports in 1988 that *Listeria*-induced chorioamnionitis is implicated [33]. Furthermore, mice infected with *LM* exhibit both systemic and local infection and related pregnancy loss [34]. The source of placental *LM* is thought to be from haematogenous seeding. Positive placental cultures can be seen in up to 76% of cases of maternal-fetal listeriosis [21], and significant evidence exists for extra-villous trophoblasts and syncitiotrophoblasts to become infected [35,36,37]. We have previously demonstrated that viral priming with a TLR3 agonist increases susceptibility to bacterial-induced inflammation via stimulation of the TLR2 by heat-killed *LM* in an in vitro model of haematogenous spread of polymicrobial infection [38]. Figure 3 shows data from this study, represented showing only the results of single treatment with heat-killed *LM*. Treatment of peripheral blood mononuclear cells and placental explants, taken from healthy pregnant women at term, revealed that heat-killed *LM* increased production of pro-inflammatory cytokines IL-1β, IL-6, IL-8, TNF-α, chemokines MIP-1α and MIP-1β, and PGE2 (Figure 3A–D). Similarly, animal models have demonstrated proinflammatory mediatory production in microglia in the brains of *Listeria* infected mice [39].

## 7. Conclusions

**Figure 4:**
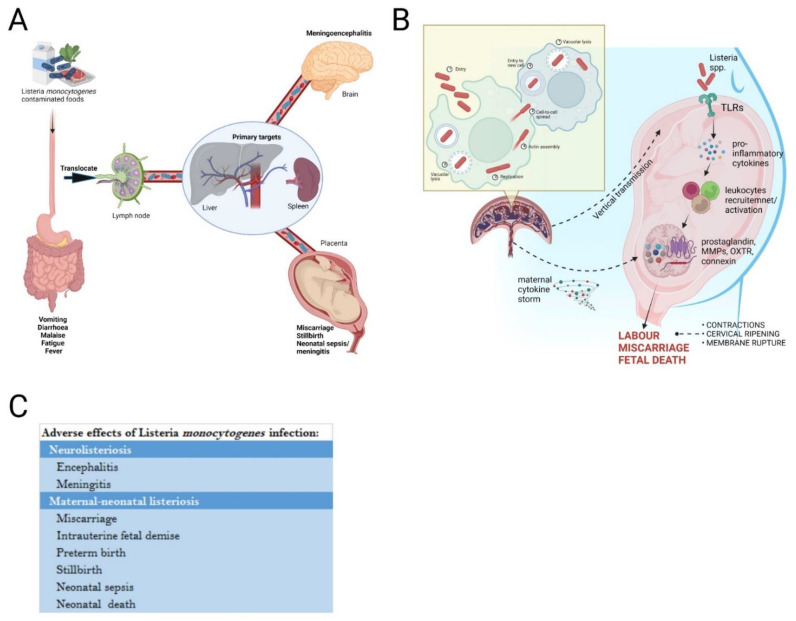
The transmission, life cycle, immune response, and adverse effects of *Listeria monocytogenes* in pregnancy. (**A**) *Listeria monocytogenes* is introduced in vivo via ingestion of contaminated foods such as dairy products, meats, and vegetables. Bacteria cross the intestinal barrier, translocate to lymph nodes to reach the primary target organs; liver and spleen where they disseminate through bloodstream to the brain and placenta causing detrimental effects. (**B**) In pregnancy, Toll Like Receptors (TLRs) expressed on the immune and endothelial cells at the fetal-maternal-interface will be activated once in contact with bacteria. This activation will cause a cascade of intracellular signaling events and the release of pro-inflammatory cytokines which will further recruit pro-inflammatory leukocytes such. Vertical transmission of the bacteria from the placenta after multiplying will create a cytokine storm thus worsening the inflammatory states of the pregnancy leading to the onset of labour, miscarriage, or fetal death. (**C**) Some of the adverse effects of *Listeria monocytogenes* infection include neurolisteriosis such as encephalitis and meningitis, and maternal-neonatal listeriosis that include miscarriage, intrauterine fetal demise, preterm birth, stillbirth, neonatal sepsis, and neonatal death. TLRs (Toll like receptors).

We have summarised the pathophysiology and adverse effects of *LM*, with particular emphasis on pregnancy, and have illustrated these in Figure 4. Pregnancy is associated with an increased susceptibility and can lead to both maternal, fetal and neonatal adverse effects. Neurolisteriosis, and particularly rhombencephalitis is rare. Diagnostic challenges exist, due to vague initial symptomology, and lack of reliable diagnostic tests. In particular, our case had a long period of time whereby symptomatology was non-specific, which led to a delay in testing for *LM*. Our key messages are firstly that a low threshold for considering listeriosis is needed in any woman presenting with headache and fever, but particularly when gastrointestinal symptoms are present. Development of any neurological abnormalities should prompt MRI to look for characteristic features of *Listeria* rhombencephalitis. Secondly, as will all pregnant women with complex acute medical problems, muti-professional team input, including but not limited to, obstetrics, neurology, infectious diseases, intensive care and neonatal teams is paramount.

## Figures and Tables

**Table 1:** Summary of investigations by day of symptoms.

Day of Symptoms	Investigation	Result
8	High vaginal swab microscopy, culture and sensitivity (MCS)	No sig growth
9	Thyroid stimulating hormone	0.98 milliunit/L
	Free T4	12.1 pmol/L
	CMV IgG blood	Not detected
	CMV IgM	Not detected
	EBV VCA IgG	Detected
	EBV VCA IgM	Equivocal
	Hep A virus IgM screen	Not detected
	Hep B virus surface Ab screen blood	Not detected
	Hep C virus Ab screen	Not detected
	HIV-1 and HIV-2 serology	Not detected
	Varicella zoster virus IgG blood	995.1 mIU/mL
	EBV EBNA-1 IgG blood	Detected
	HEV RNA detection	Not detected
	SARS-CoV-2 virus RNA	Not detected
11	Ferritin	137 µg/L
	Immunoglobulin A level	0.96 g/L
	Immunoglobulin G level	8.1 g/L
	Immunoglobulin M level	0.48 g/L (low)
	Protein electrophoresis	No abnormalities detected
	ANA	Negative
	ANCA screen	Negative
	*Borrelia burgdorferi* Ab level blood	Not detected
	Respiratory Virus PCR screen (including Influenza A virus, Influenza A subtyping, Influenza B virus, Respiratory Syncytial virus PCR, rhinovirus/enterovirus, parainfluenza 1, 2, Parainfluenza 4 virus, adenovirus PCR, HMPV PCR, Bordetella species, Bordetella pertussis, mycoplasma pneumoniae)	Not detected
12	Lumbar puncture	Glucose CSF 2.1 mmol/L, lactate 3.8 mmol/L, protein 1.88 g/L
	CSF MCS	75 WBCs, RBCs 1, differential 80% mononuclear, 20% polymorphs. Gram stain no organisms seen and no growth at 2 days
	CSF measles virus RNA, mumps virus RNA, listeria monocytogenes DNA	Not detected
	Syphilis serology	Not detected
14	LGI1 Ab blood	Negative
	Neuronal Ab level serum	Neuronal IIF screen Hu/ANNA-1 Ab neg Yo/PCA-1 Ab neg Ri/ANNA-2 Ab neg GAD Ab neg
	Anti-NMDA receptor Ab level	Negative
	CASP2 Ab blood	Negative
	West Nile virus IgM, *Borrelia burgdorferi* IgG/IgM, Leptospirosis IgM, Sandfly fever Sicilian virus IgG, Sandfly fever Naples virus IgG, Sandfly fever Toscana virus IgG, Sandfly fever Cyprus Virus IgG	Negative
	SARS-CoV-2 N protein Ab blood	Not detected
	*Chlamydia psittaci* serology	IgG antibodies < 64
	*Coxiella Burnetii* Pneumonia	Negative
	Syphilis serology blood	Not detected
	*Mycoplasma pneumoniae* IgG serology PCR	negative
15	Vitamin D level	41.2 nmol/L (low, range 70–150)
	Vitamin B12 level	594 ng/L
	Folate	11.8 µg/L
	Cortisol	1568 nmol/L
16	Lumbar puncture	Glucose 2.7 mmol/L, lactate 3.2 mmol/L
	Cerebrospinal fluid MCS	111 WBCs/cmm, 10 RBCs/cmm, 100% mononuclear cells Gram stain no organisms seen and no growth after extended incubation
	Virus screen on CSF including HSV-1, HSV-2, varicella zoster virus, cytomegalovirus, Epstein–Barr virus, enterovirus, parechovirus, HHV-6, *Listeria monocytogenes* DNA, measles virus RNA, Mumps virus RNA	Not detected
	Oligoclonal bands CSF	CSF IgG is not oligoclonal. Results indicate that local synthesis of IgG is unlikely
	Glutamic acid decarboxylase Ab (GAD) CSF	Negative
	Borrelia PCR CSF	Negative
	Cryptococcus Ag screen CSF	Negative
	Acanthamoeba CSF	Not isolated
	CSF Auramine stain	No growth at 6 weeks, no acid alcohol fast bacilli seen
	Meningococcal screening PCR test	negative
17	Aspergillus Galactomannan Ag	Negative
	Brucella species Ab level blood	Negative
	Cryptococcal Ag	Negative
	Histoplasma species Ab level blood	negative
	Toxoplasma species screen blood	Negative
	Beta Glucan Beta D-glucan antigen test	Negative with concentration < 30 pg/mL
18	Lumbar puncture	Glucose CSF 2.9 mmol/L, protein 0.99 g/L
	CSF MCS	88 WBCs/cmm, 280 RBCs/cmm. Polymorphs 10%, mononuclear cells 90%. Gram stain no organisms seen and no growth at 2 days
	CSF cytology	Extremely cellular and consists of mature lymphoid cells in large numbers. Occasional neutrophils and monocytes are present which could possibly be blood derived. Features suggest a lymphocyte rich inflammatory process. No malignant cells seen. Final diagnosis lymphocytic inflammation
	LGi1 Ab CSF	Negative
	NMDA receptor Ab CSF	Negative
	CASP2 Ab CSF	Negative
	Long term fungal culture CSF	No fungi isolated after 7 days, 2 weeks and 4 weeks incubation
	TB PCR CSF	Negative CSF
	Cryptococcus Ag screen CSF	negative
19	Amylase	92 unit/L
	Absolute lymphosum count	875 cells/µL (low, range 1000–2800)
	CD3+ lymphocytes % blood	69.7%
	CD3+ lymphocytes absolute blood	610 cells/µL (low, range 700–2100)
	CD4:CD8 ratio	1.9
	CD4+ lymphocytes % blood	45.1%
	CD4+ lymphocytes absolute blood	384 cells/µL
	CD8+ lymphocytes %	24.3
	CD8+ lymphocytes absolute blood	207 cells/µL
	CD56+ lymphocytes % blood	10.5
	CD56+ lymphocytes absolute blood	95 cells/µL
	Total B lymphocyte count	183 cells/µL
	B lymphocytes % blood	20.3% (high, range 6–19)
	IgG subclass 1 level	2.05 g/L (low, range 3.2–10.2)
	IgG subclass 2 level	2.36 g/L
	IgG subclass 3 level	0.29 g/L
	IgG subclass 4 level	0.18 g/L
27	Hepatitis B virus surface Ag blood	Not detected
29	Smooth muscle Ab level	Negative
	Liver and kidney microsomal Ab	Negative
	Mitochondrial Ab level	Negative
	Gastric parietal cell Ab level	Negative
8, 11	Blood cultures	No growth
24, 29, 35	Carbapenem Res Orgs screen culture rectal swab	No carbapenem resistant organisms isolated
15, 20, 24, 29, 35	MRSA nose and groin screen	MRSA not isolated
11, 15	Throat swab	No significant growth
9, 11, 25, 30, 31, 38	Urine culture	No growth
11, 25, 30, 31, 32, 38	Urine microscopy	WCC < 50 cmm, RBC not seen, scanty epithelial cells

## Data Availability

The data presented in this study are available on request from the corresponding author.
